# Currents in Dental Sleep Medicine

**DOI:** 10.11607/ofph.2023.3.ar

**Published:** 2023-11-17

**Authors:** Dennis Bailey, Takafumi Kato

Since the comorbid and bidirectional relationship between sleep apnea and insomnia was first recognized in 1973, there has been the need to further recognize that these two disorders frequently occur together and may have an impact on one another. In 1999 and 2001, further recognition that these two disorders mutually impacted one another was better defined, and it was decided that the high prevalence of their co-existence needed to be addressed. Thus, the term *COMISA* was established in 2017, and there has been more research on this relationship over the the last 5 years.

It is well recognized that insomnia and obstructive sleep apnea are the most prevalent sleep disorders. Insomnia is mainly self-reported and needs to be present for more than 3 months to be considered chronic. The co-occurrence of insomnia and sleep apnea is well established. In those with sleep apnea, 30% to 50% have symptoms of insomnia, and in those with insomnia, 30% to 40% have sleep apnea. It is well recognized that many of the health-related consequences of sleep apnea may also exist in those with insomnia alone; however, in the presence of COMISA, many of these conditions are worse overall. This relates to physical and mental health, quality of life issues, and sleep quality.

As the article referenced from the Sleep Heart Health Study, it was determined that there was a 47% increased risk for all-cause mortality and a 75% increase in prevalence of cardiovascular disease when COMISA is present. These findings were impacted by the presence of depression, which is directly related to poor sleep such as that found in people with insomnia or sleep apnea and made worse by COMISA. Many of these findings have been shown to impact adherence to continuous positive airway pressure (CPAP) therapy, thus worsening the impact of these conditions. It was found that adherence to PAP therapy was approximately 30% less in COMISA patients compared to those who had only sleep apnea. It can also be assumed that this could also apply to oral appliance therapy, an avenue to be explored.

A bidirectional relationship is present here. It is now becoming recognized in sleep medicine that sleep apnea can directly affect insomnia symptoms, even though some symptoms of insomnia may improve with PAP therapy. In a similar manner, insomnia may impact sleep apnea by virtue of increased arousals and the lack of stable sleep along with modification of the arousal threshold. A low arousal threshold has been shown to be a factor in the inadequate management and resolution of sleep apnea. Recognition of COMISA should be managed as well. The most effective way to do so is with cognitive behavioral therapy targeting insomnia (CBT-I), which has been shown to assist with the management of co-occurring sleep apnea. It was reported that the Apnea-Hypopnea Index (AHI), based on home sleep testing, was appreciably reduced over a 6-week period when CBT-I was employed, and PAP adherence was also improved, as was the frequency of awakenings. Given that the co-occurrence of insomnia with sleep apnea is now well recognized, the approach to both its recognition and management needs to be employed along with the standardized approach used for sleep apnea. This includes the introduction of screening forms such as the Insomnia Sleep Severity questionnaire and online programs for CBT-I.

The importance of this relationship in orofacial pain practice is significant. It is well established that there is also a bidirectional relationship between pain and poor sleep, with poor sleep contributing to pain exacerbation, such as that found in patients with insomnia or sleep apnea alone. If it is determined that a patient with pain is at risk for COMISA, then management of the pain may be more difficult. And, finally, should management directed at insomnia or sleep apnea alone result in less than adequate outcomes, the possible presence of COMISA needs to be considered. Thus, interprofessional collaboration is needed more than ever.

Insomnia and obstructive sleep apnea (OSA) are both highly prevalent sleep disorders. It is known that insomnia can co-occur with other conditions (eg, sleep-related periodic limb movement, sleep bruxism, pain, anxiety, and depression). Insomnia co-occurrence was defined by the International Classification of Sleep Disorders Revised 2023 version^1^ as:

- Comorbid insomnia: An insomnia disorder associated with, but partially or totally independent of, a co-occurring sleep disorder.
- COMISA: Comorbid (CO) insomnia (I) sleep apnea (SA). This sleep disorder happens when insomnia and OSA co-occur and was reported by Guilleminault et al in 1973.^2^

Interestingly, insomnia and OSA may also have bidirectional relationships with chronic orofacial pain conditions, including temporomandibular disorders (TMDs).^3,4^ However, there is a limited amount of basic research that can conclusively demonstrate the strong connection and causality between these sleep disorders and orofacial pain. Recent research using sophisticated animal models has aimed to clarify the potential involvements of stress-induced insomnia and OSA in the onset of orofacial pain.

The first study, conducted by Dalanon et al, aimed to investigate the impact of predictable chronic mild stress (PCMS) on both sleep-wake patterns and pain thresholds in male mice. PCMS was administered using mesh wire (MW) and water (W) rearing conditions. MW mice were reared on wire netting on top of regular sawdust, while W mice were placed in a cage with water positioned 2 mm below the wire netting for a duration of 21 days. Sleep recordings were conducted on both day 2 and day 21. The findings revealed that exposure to PCMS reduced nonrapid eye movement (NREM) sleep during the dark period, a time when animals are typically active. Importantly, PCMS diminished slow-wave activity, which serves as a cortical indicator of deep sleep, during NREM sleep in both groups during both the light and dark periods. These findings suggest that PCMS led to a decrease in sleep quality. Orofacial pain–related behavior was assessed by observing the behavioral response to thermal (cold or hot) stimulation on the buccal skin. Increased pain sensitivity to cold stimuli was observed in both the MW and W groups on day 21. PCMS also heightened mechanical and aversive hot thermal hypersensitivity in the tails and paws of mice. Additionally, mice subjected to PCMS exhibited elevated plasma corticosterone levels, indicating increased stress levels. Thus, the PCMS model resulted in reduced sleep quality and potentially heightened nociceptive hypersensitivity in both the orofacial and limb regions.

The second study, by Kishimoto et al, investigated the impact of chronic intermittent hypoxia (CIH) during the light period (when rodents predominantly sleep) on intraoral and ocular sensations in rats. Male rats were housed in an experimental chamber and exposed to CIH for 6 hours a day for 16 days. The intermittent hypoxia protocol comprised alternating cycles, with periods of hypoxia reaching as low as 5% O_2_, followed by 3 minutes of exposure to N_2_, and then 3 minutes of returning to normal oxygen levels. Intraoral pain sensation was assessed using a two-bottle test to measure capsaicin solution preference. CIH-exposed rats exhibited increased capsaicin sensitivity, indicated by decreased consumption of the capsaicin solution, starting from 3 days after the beginning of CIH until day 16. Additionally, sensitivity to capsaicin on the ocular surface was enhanced. The number of TRPV1-positive neurons, especially larger ones, increased in the trigeminal ganglion of CIH-exposed rats. In the trigeminal spinal subnucleus caudalis (Vc), there was a higher density of TRPV1-positive nerve terminals. When low-dose capsaicin was applied to the tongue under anesthesia, the number of c-fos positive cells was significantly higher in the Vc of CIH-exposed rats. However, when the hypoxic condition was switched to normal oxygen conditions after day 8 of hypoxia, the aforementioned behavioral and immunohistochemical responses were reversed by day 16. Overall, the study suggests that sleep apnea–related CIH could temporarily heighten orofacial pain sensitivity.

These two translation-type animal studies support that, in humans, stress-related insomnia and OSA could enhance orofacial pain sensitivity. When interpreting these findings, it is crucial to acknowledge methodological limitations. Dalanon et al, the causal links between chronic stress, reduced sleep quality, and heightened pain sensitivity may not be fully understood. In Kishimoto et al, it is unclear if intermittent hypoxia or secondary sleep disruptions trigger pain sensitivity. Both studies were performed in young male animals, and therefore age and sex should be taken into consideration in future research. Further research is also needed to determine whether these animal translational models also impact musculoskeletal pain conditions like temporomandibular disorders (TMDs), which will help guide us to further development in management.

In recent years, cases of comorbid insomnia and OSA, referred to as COMISA, have highlighted its complex pathophysiology. In chronic orofacial pain patients with COMISA, the insomnia, OSA, or another co-occurring condition may contribute to specific pathophysiologic profiles independently or together.
